# Correction to: Cellular immunotherapy using irradiated lung cancer cell vaccine co-expressing GM-CSF and IL-18 can induce significant antitumor effects

**DOI:** 10.1186/s12885-020-6544-x

**Published:** 2020-01-17

**Authors:** Hongwei Tian, Gang Shi, Guoyou Yang, Junfeng Zhang, Yiming Li, Tao Du, Jianzhou Wang, Fen Xu, Lin Cheng, Xiaomei Zhang, Lei Dai, Xiaolei Chen, Shuang Zhang, Yang Yang, Dechao Yu, Yuquan Wei, Hongxin Deng

**Affiliations:** State Key Laboratory of Biotherapy, West China Hospital, Sichuan University, Chengdu, Sichuan 610041 The People’s Republic of China

**Correction to: BMC Cancer (2013) 14:48**

**https://doi.org/10.1186/1471-2407-14-48**

Following publication of the original article [1], the authors reported an error in Fig. 5 of this article, graphs presenting FCM and immunofluorescent for CD4T, CD8T and NK cell of the Control Groups (LL2, LL2-irradation, MCS-irradiation) were inadvertently duplicated from another parallel experiment. The correct version of this figure appears below. To the best of our knowledge, this does not affect the conclusions. We apologize for the error.

**Fig. 5:**
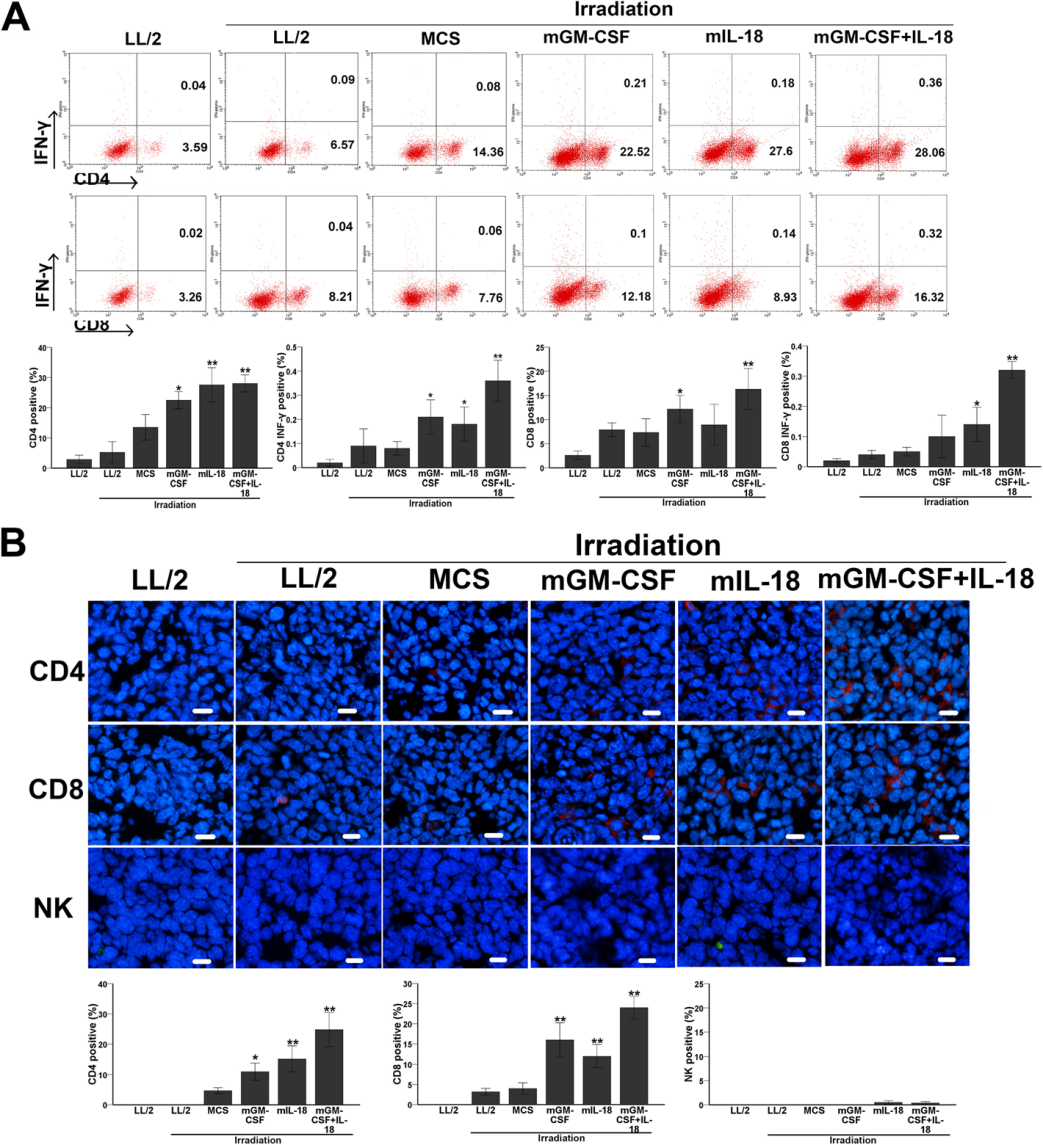
Increased proliferation of CD4 ^+^ INF-γ ^+^ T, CD8 ^+^ INF-γ ^+^ T in spleen and infiltration of CD4 ^+^ T, CD8 ^+^ T in tumors. Spleen lymphocytes were isolated and stained for CD4, CD8 and INF-γ double staining antibodies by flow cytometry; Tumor tissue was obtained 3 days after the last measurement of tumor volume, frozen sections were used for analysis of CD4, CD8 T and NK cell infiltration. **a** The proportion of CD4^+^INF-γ^+^ T, CD8^+^ INF-γ^+^ T in co-expression IL-18 and GM-CSF-treated mice was significantly higher than control groups (*P* < 0.01, *n* = 7). Experiments were performed in triplicate and repeated three times. **b** Immunofluorescence staining of tumor tissue with CD4, CD8 and NK antibody showed that CD4^+^, CD8^+^ T cell infiltrations was significant enhanced in co-expression IL-18 and GM-CSF-treated group as compared with control groups (*P* < 0.01, *n* = 7) (original magnification, ×200)

## References

[1] Tian H (2013). Cellular immunotherapy using irradiated lung cancer cell vaccine co-expressing GM-CSF and IL-18 can induce significant antitumor effects. BMC Cancer.

